# Connectivity of the Frontal Cortical Oscillatory Dynamics Underlying Inhibitory Control During a Go/No-Go Task as a Predictive Biomarker in Major Depression

**DOI:** 10.3389/fpsyt.2020.00707

**Published:** 2020-08-03

**Authors:** Ying-lin Han, Zhong-peng Dai, Mohammad Chattun Ridwan, Pin-hua Lin, Hong-liang Zhou, Hao-fei Wang, Zhi-jian Yao, Qing Lu

**Affiliations:** ^1^Department of Psychiatry, The Affiliated Brain Hospital of Nanjing Medical University, Nanjing, China; ^2^School of Biological Sciences & Medical Engineering, Southeast University, Nanjing, China; ^3^Key Laboratory of Child Development and Learning Science, Ministry of Education, Southeast University, Nanjing, China; ^4^Medical School of Nanjing University, Nanjing Brain Hospital, Nanjing, China; ^5^Department of Psychology, Jiangsu Province Hospital Affiliated to Nanjing Medical University , Nanjing, China

**Keywords:** cognitive inhibition, functional connectivity (FC), go/no-go task, magnetoencephalography (MEG), major depressive disorder (MDD), power spectrum (PS)

## Abstract

**Background:**

Major depressive disorder (MDD) is characterized by core functional deficits in cognitive inhibition, which is crucial for emotion regulation. To assess the response to ruminative and negative mood states, it was hypothesized that MDD patients have prolonged disparities in the oscillatory dynamics of the frontal cortical regions across the life course of the disease.

**Method:**

A “go/no-go” response inhibition paradigm was tested in 31 MDD patients and 19 age-matched healthy controls after magnetoencephalography (MEG) scanning. The use of minimum norm estimates (MNE) examined the changes of inhibitory control network which included the right inferior frontal gyrus (*r*IFG), pre-supplementary motor area (preSMA), and left primary motor cortex (*l*M1). The power spectrum (PS) within each node and the functional connectivity (FC) between nodes were compared between two groups. Furthermore, Pearson correlation was calculated to estimate the relationship between altered FC and clinical features.

**Result:**

PS was significantly reduced in left motor and preSMA of MDD patients in both beta (13–30 Hz) and low gamma (30–50 Hz) bands. Compared to the HC group, the MDD group demonstrated higher connectivity between *l*M1 and preSMA in the beta band (*t* = 3.214, *p* = 0.002, FDR corrected) and showed reduced connectivity between preSMA and *r*IFG in the low gamma band (*t* = −2.612, *p* = 0.012, FDR corrected). The FC between *l*M1 and preSMA in the beta band was positively correlated with illness duration (*r* = 0.475, *p* = 0.005, FDR corrected), while the FC between preSMA and *r*IFG in the low gamma band was negatively correlated with illness duration (*r* = −0.509, *p* = 0.002, FDR corrected) and retardation factor scores (*r* = −0.288, *p* = 0.022, uncorrected).

**Conclusion:**

In this study, a clinical neurophysiological signature of cognitive inhibition leading to sustained negative affect as well as functional non-recovery in MDD patients is highlighted. Duration of illness (DI) plays a key role in negative emotional processing, heighten rumination, impulsivity, and disinhibition.

## Introduction

Major depressive disorder (MDD) is a debilitating disease which is linked to persistent episodes of low mood, anhedonia, and prominent deficits in high-order executive function. In MDD patients, emotion regulation lies at the heart of inhibitory control, attentional biases, rumination, impulsivity, and mood-congruent materials (1).

MDD patients demonstrate minimal cognitive changes in the early stage, and clinical symptoms appear to worsen as the disease progresses (2). Cognitive deficits provoke profound functional disability and deteriorate quality of life as well as reducing educational, occupational, and social outcomes (3). Although cognitive dysfunction is highly correlated with psychosocial functioning, there is also a detrimental synergy between failure of behavioral inhibition and social cognition (4). MDD patients who are living with cognitive control deficits tend to present with poorer functional outcomes and to pose a high risk for psychosis. It has been suggested that discrepancies in cognitive inhibition lead to a heightened vulnerability to ruminative responses, negative mood states, and memory impairment in people with depression (5). Additionally, mood-congruent cognition is usually transient and is rapidly replaced by thought or memory while attempting to regulate and repair negative moods (1).

Motor inhibition, a fundamental component of executive control, enables us to rapidly cancel motor activity even after its initiation, thus actively suppressing a movement due to environmental demands (6). In affective disorders, it has been ascertained that inhibitory dysfunction stems from a confusion in the orbitofrontal, prefrontal, insular, and temporal cortices, as well as the amygdala and striatal brain regions (7–10). In mood disorders, in which levels of cognitive impairment are extremely severe, structural abnormalities in orbital and medial frontal regions as well as in the temporal lobe will be seen (11–13). Moreover, the mechanisms in which inhibited behaviors manifest in affected brain regions are vital for understanding the performance of disinhibition *via* motor circuits. The neural substrate underlying the successful response inhibition involves in the right inferior frontal gyrus (*r*IFG) (14), the pre-supplementary motor area (preSMA), and left primary motor cortex (*l*M1) (6, 15, 16).

Electrophysiological measurements can provide a temporally precise estimate of oscillatory dynamics in the context of cognitive and behavioral tasks. The use of such evidence suggests the significance of frequency-specific function connectivity between prefrontal, premotor, and motor cortex in the beta (12–30 Hz) (17) and gamma ranges (>30 Hz) (18). In several neurological and psychotic diseases, beta power is both significantly transformed and characteristic for motor control (19, 20). Beta oscillations are crucial in feedback interactions between the IFG and motor areas, especially during response inhibition (17). Additionally, alterations in gamma power are observed during action control (18). Measurable changes in frequency-specific bandwidths may provide the key to the mechanistic link between the neuropathological specificity of depression and impaired behavior. Currently, it is unclear whether an altered pattern of activities occurs during response inhibition *via* modulation of specific frontal network intercortical inhibition or due to withdrawal of facilitation.

In this study, we used MEG during a task of response inhibition to examine the effect of depression on frequency-specific changes concerning behavior as well as connectivity between prefrontal, premotor, and motor cortex. A go/no-go task was utilized to assess inhibitory control (21, 22). This computerized test can elicit prepotent motor activity (“go”) which sometimes has to be inhibited (“no-go”) (23, 24). To measure network connectivity and quantify the parameters, we used formal measures of evidence from specific models of frontal brain networks (5, 17).

**Figure 1:**
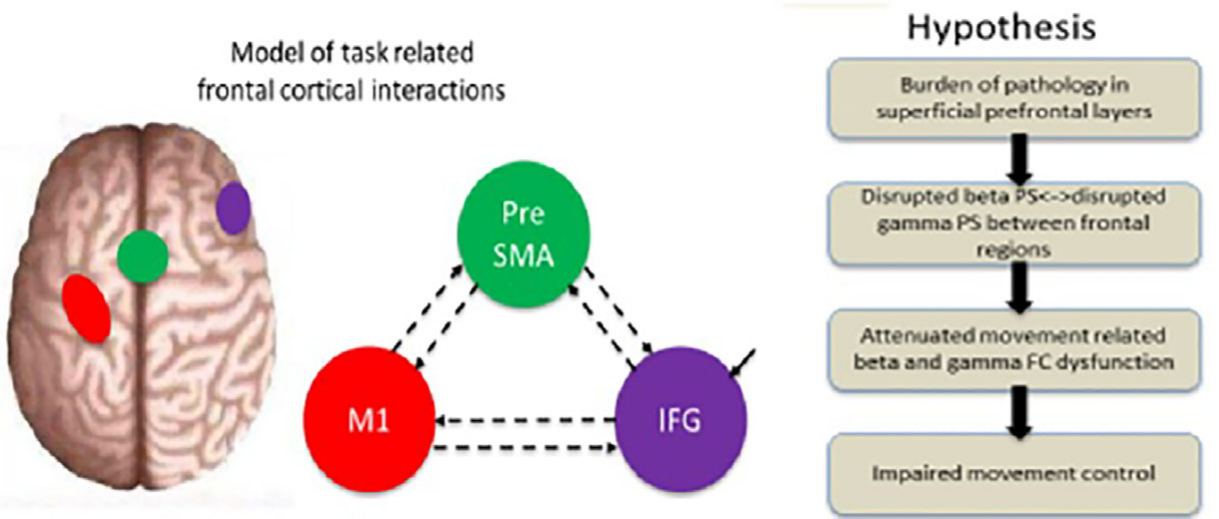
Illustration of the experimental background and principal hypothesis. A model of the regions included in the dynamic analysis (rIFG, preSMA, and lM1) and the specified connectivity. Hypothesis: the layerspecific burden of pathology is predicted to disrupt the specific-frequency, attenuate dysfunction in the beta and gamma bands, and consequently impair movement control. Modified from (32).

The altered patterns of PS and FC are associated with cognitive deficit severity in depression (25, 26). Connections between *l*M1, preSMA, and *r*IFG have been widely demonstrated to be associated with inhibition mechanisms (6, 14–16). MDD show abnormal PS in the beta and gamma bands compared to healthy controls (HC) (27, 28). Authors of previous studies have demonstrated that the FC and PS in the beta and gamma bands among these regions may be a strong indicator of cognitive deficit in depression (25, 26, 29–31). However, the relationship between FC and inhibition deficit outcome is unclear, particularly with respect to FC in high-frequency bands (≥13 Hz). We predicted a clinically meaningful inhibition of the FC and PS patterns in the beta and gamma bands in MDD patients. Additionally, the FC in the beta and gamma bands could be altered among frontal cortical network regions for behavioral control in depression. The hypotheses could delineate the significance of cognitive dysfunction as a symptomatic target for prevention and treatment of MDD (**Figure 1**).

## Materials and Methods

### Participants

A total of 50 participants, including 31 MDD medication-naïve patients and 19 HC, were recruited to perform a go/no-go task during MEG scanning. MDD patients were enrolled from both the outpatient and inpatient psychiatry departments at the Affiliated Brain Hospital of Nanjing Medical University. HC were enlisted *via* advertisements in the same area. All participants were right-handed. MDD diagnosis was confirmed by a psychiatrist using the Structured Clinical Interview for DSM-IV Axis I Disorders (SCID) as well as the International Statistical Classification of Diseases and Related Health Problems 10^th^ Revision (ICD-10). MDD severity was assessed using the 17-item Hamilton Rating Scale for Depression (HRSD-17).

Inclusion criteria for MDD participants were as follows: (1) aged between 18 and 45; (2) a diagnosis of MDD based on the DSM-IV and ICD-10; (3) a total HRSD-17 score of >24; (4) no psychotropic treatments, including anti-depressants, mood stabilizers, antipsychotics, and benzodiazepines for the past 2 weeks; (5) no physical therapy, such as modified electro-convulsive therapy (MECT) or repetitive transcranial magnetic stimulation (RTMS) for the past 6 months; (6) a score of <5 on the Young Mania Rating Scale (YMRS).

Patients who met any of the following criteria were excluded from the study: (1) serious medical conditions such as organic brain disorders and severe somatic disease, as assessed by past medical history or laboratory analysis; (2) history of substance abuse; (3) family history of any psychiatric disorders except MDD; (4) pregnant or lactating women; (5) contraindications for MEG or MRI.

HC were examined using the Structured Clinical Interview for DSM-IV Axis I Disorders-Research Version-non-Patient Edition (SCID-I/NP). Exclusion criteria for this group were as follows: (1) previous manic or hypomanic episode; (2) any neurological, psychiatric, or endocrine illnesses; (3) family history of major psychiatric disorders in first degree relatives; (4) history of substance abuse; (5) any serious physical illness, such as cardiovascular diseases, infectious diseases, tumor, or so on, as evaluated by laboratory analysis or history; (6) history of psychiatric illnesses; (7) pregnant or lactating women; (8) contraindications for MEG or MRI.

### Go/No-Go Task

**Figure 2:**
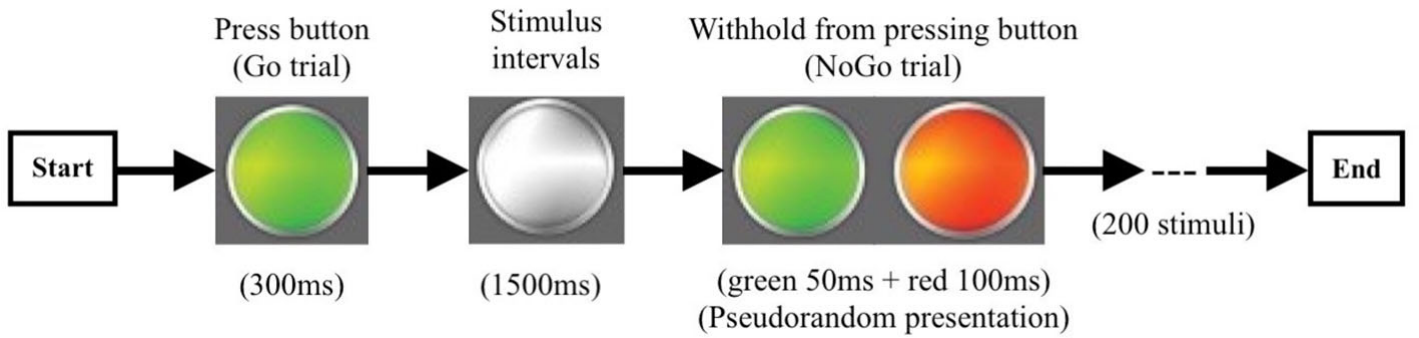
The procedure of the go/no-go experiment. Go trail (green light): 300 ms/per stimulus, 150 times; No-go trail (green + red light): 150 ms/per stimulus, 50 times; Stimulus Intervals 1,500 ms.

The go/no-go task was completed during MEG scanning after recruitment. This task was depicted in **Figure 2**. It consisted of randomly displayed visually cued tasks, including 150 go trials and 50 no-go trials. Stimuli were controlled using BrainX ^®^. Each trial started with a white light presented centrally on a gray background for 1,500 ms, followed by a color cue that subtended 20°. Go trials were cued with a “long green” light presented centrally until the response button was pressed or for 300 ms if there was no response. No-go trials were cued with “short green (50 ms) + short red (100 ms)” lights which were displayed for 150 ms. Trials order was pseudo-random and permuted such that on 15% of trials, a no-go cue was shown after three, five, and seven go trials, and on 10% of trials, a no-go cue was displayed after two, four, and six go trials. Participants were instructed to focus on the white light and press the response button with their right hand as quickly as they could every time the go cue appeared and not to press the button when the no-go cue was shown. Before the MEG recordings, all participants undertook 30 practice trials to confirm whether they had understood the task.

### MRI Image Acquisition

All participants were scanned with a Siemens Verio 3T MRI system using a high-resolution, T1-weighted, 3D gradient-echo pulse sequence (TR = 1,900 ms, TE = 2.48 ms, FA = 9°, slice number = 176, slice thickness = 1 mm, voxel size = 1 × 1 × 1 mm^3^, FOV = 250 × 250 mm^2^). To ensure the offline co-registration of MRI and MEG, three fiducial markers were placed on the nasion as well as the left and right pre-auricular.

### MEG Image Acquisition

MEG data were recorded with an Omega 2000, 275 channels whole-head CTF MEG system (VSM Med Tech Inc., Port Coquitlam, Canada) at a sampling rate of 1,200 Hz in a magnetically shielded room. Recordings lasted 10 min. Head coils were placed at the nasion as well as the left and right pre-auricular points to localize the head position. The locations of the fiducial markers and MEG sensors were confirmed with respect to brain anatomy by matching the digitized head surface to the head surface extracted from anatomical MRI.

### MEG Data Analysis

MEG data were pre-processed using a band-pass offline filtering (1–100 Hz) to (with removing) remove power-line interference (50 Hz). Artifactual epochs (that was, eye movements and strong muscle activity) were removed following visual inspection. Processed MEG data were frequency filtered into full-band (1–80 Hz) waves, including theta (4–8 Hz), alpha (8–13 Hz), beta (13–30 Hz), low gamma (30–50 Hz), and high gamma (50–80 Hz). Neural data in sensor level was projected onto source space with a 6 mm grid using a MNE method (31, 33, 34). Spatial filters with the axial gradiometer data at each grid point were multiplied across the entire brain to obtain source activities. After acquiring the time-series (source activities), a power envelope correlation was employed. The Montreal Neurological Institute (MNI) coordinates of the left motor cortex (-37,-25,64), preSMA (-4,4,60), and right IFG (48,18,-2) identified according to previous research findings (35, 36). Then the signal of regions of interest (ROI) was extracted within a 6 mm sphere centered on MNI coordinates.

### Statistical Analysis

PS method: power spectral density represents the amount of energy described by a time series when transformed into a spectral function (37):

$$P\left(\omega \right)=\underset{T\to \infty }{lim}\frac{1}{T}{\left|F_{T}\left(\omega \right)\right|}^{2}$$

FC method: orthogonalization time-point by time-point, which requires no assumption about the stationary signals' relation beyond the length of the carrier-frequency dependent analysis window (38):

$$Y_{⊥X}\left(t,f\right)=imag\left(Y\left(t,f\right)\frac{X{\left(t,f\right)}^{*}}{\left|X\left(t,f\right)\right|}\right)$$

Two-sample *t*-test were performed to compare differences in the PS and FC among the ROIs in each band between the MDD and HC groups (survived FDR correction with a threshold *p* value 0.05).

The FC (between *l*M1 and preSMA, between *l*M1 and *r*IFG, between preSMA and *r*IFG) in each specific frequency band, clinical information (length of disease, family history, education level), total HRSD-17 score, and each HRSD-17 factor score (anxiety/somatization, cognition, weight, sleep, retardation) were analyzed using Pearson correlation. We used Statistical Package for the Social Sciences version 24 software (SPSS, IBM). Significance was set at *p* < 0.05 and all statistical tests were two-tailed.

## Results

### Demographic and Clinical Characteristics

**Table 1** contains the demographic and clinical characteristics of the two groups. HRSD-17, illness duration and family history were recorded for patients only. Details are summarized in **Table 1**.

**Table 1:** Demographic and clinical characteristics of the sample.

	MDD n = 31	Controls n = 19	t/X^2^	*p* value
Gender (male/female)	16/15	10/9	0.199^a^	0.655
Age (years)	31 ± 8.47	31.53 ± 7.40	0.054^b^	0.988
Education (years)	13.52 ± 2.91	13.24 ± 2.46	0.103^b^	0.838
Handedness (right/left)	31/0	19/0		
Outpatient/Inpatient	10/21			
HRSD-17	30.27 ± 6.84			
Duration of illness (months)	13.85 ± 13.11			
Family history	11(+)/20(−)			

Data are presented as the range of mean ± standard deviation (two-sample t-test).

*p < 0.05. ^a^Chi-square test, ^b^Two-sample t-test.

### Behavior

Behavioral analysis was conducted to examine the mean reaction time for correct go and incorrect no-go responses, while response accuracy was measured using a two-sample *t*-test. Reaction time and accuracy rates can be seen in **Table 2**. Compared to the control group, the MDD group had significantly longer reaction time (*p* = 0.041) and lower accuracy (*p* = 0.042) when responding to no-go task. There were no statistically significant behavior differences between groups in go task. Therefore, the changes in specific frequency activity between groups at no-go task were scrutinized.

**Table 2:** Mean reaction times and accuracy rates for go and no-go trials.

	MDD	Controls	*p* value
**Reaction times (ms)**			
Go	200.83 ± 7.84	188.44 ± 7.89	0.948
No-Go	509.23 ± 13.96	356.75 ± 39.28	0.041^*^
**Accuracy (%)**			
Go	100%	100%	–
No-Go	96.00% ± 3.39	98.33% ± 1.63	0.042^*^

*p < 0.05.

### Differences of PS at No-Go Trials

The changes of PS in full-band between groups were assessed throughout the procedure as described in *Methods*. Interestingly, during the no-go task, the significant down-regulated PS in *l*M1 and preSMA were concentrated in the beta and gamma bands for the MDD group, while there were no statistically significant differences of PS in the theta and alpha bands between groups (**Table 3**).

**Table 3:** Discrepancy of PS in theta ~ gamma frequency between MDD and HC groups.

	Significant regions	MDD	Controls	*t-*value	*p*-value
Theta	*l*M1	4.50 ± 4.54	7.81 ± 6.29	−2.162	0.076
(4–7Hz)	preSMA	6.35 ± 6.71	6.60 ± 5.18	−0.143	0.524
Alpha	*l*M1	4.92 ± 4.70	8.14 ± 6.37	−2.053	0.126
(8–13Hz)	preSMA	6.24 ± 5.37	5.32 ± 3.87	0.647	0.118
Beta	*l*M1	8.30 ± 5.48	16.25 ± 13.12	−3.023	0.000^***^
(13–30Hz)	preSMA	3.03 ± 2.31	6.73 ± 6.58	−2.925	0.001^***^
Low gamma	*l*M1	7.96 ± 5.45	15.39 ± 12.60	−2.930	0.001^***^
(30–50Hz)	preSMA	3.03 ± 2.38	6.06 ± 5.81	−2.614	0.004^**^
Gamma	*l*M1	8.16 ± 5.79	16.17 ± 14.09	−2.853	0.002^**^
(30–80Hz)	preSMA	3.11 ± 2.45	6.23 ± 5.97	−2.619	0.006^*^
High gamma	*l*M1	8.29 ± 6.07	16.67 ± 15.23	−2.782	0.004^**^
(50–80Hz)	preSMA	3.16 ± 2.51	6.34 ± 6.13	−2.604	0.008^*^

Two-sample t-test, two-sided, alpha-level 0.05, FDR corrected.

*p lt; 0.05, **p lt; 0.005, ***p lt; 0.001.

#### Differences of PS in the Beta Band (13–30 Hz)

Significant differences of PS were found in *l*M1 and preSMA between groups but not in *r*IFG. Compared to the HC group, MDD patients showed significantly reduced activity between *l*M1 and preSMA in the beta band (**Table 3**).

#### Differences of PS in All Gamma-Band (30–80 Hz, 30–50 Hz, 50–80 Hz)

Significant differences of PS were found between groups in all gamma-band Hz (including 30–80 Hz range, 30–50 Hz range, and 50–80 Hz range). Compared to the MDD group, The HC group exhibited significantly increased PS in *l*M1 and preSMA (**Table 3**).

### FC at No-Go Trials

#### Differences of FC in the Beta Band at No-Go Trials

**Figure 3:**
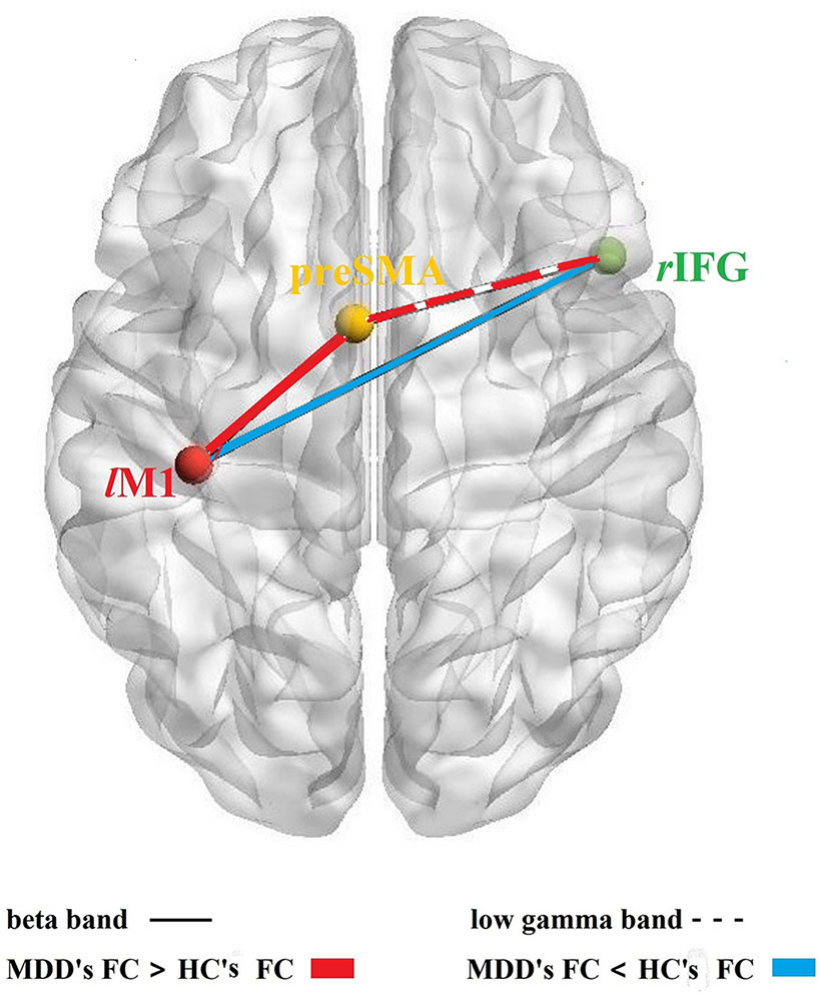
FC among *r*IFG-preSMA-*l*M1. Schematic diagram of the FC among the three core brain regions based on response inhibition network; solid lines: the FC in beta band, dotted line: the FC in the low gamma band; red color: MDD's FC > HC's FC, blue color: MDD's FC < HC's FC.

**Figure 4:**
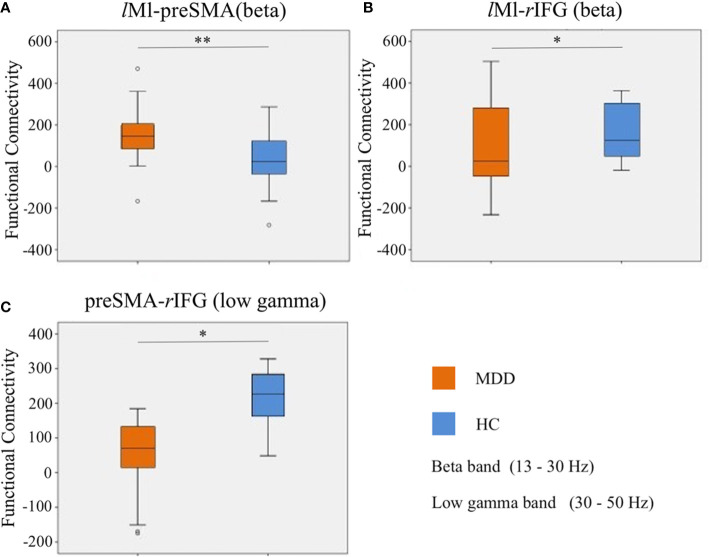
Differences of FC in the beta and low gamma bands at no-go trials. **(A)**: the FC between *l*M1 and preSMA in the beta band; **(B)**: the FC between *l*M1 and *r*IFG in the beta band; **(C)**: the FC between preSMA and *r*IFG in the low gamma band. *p < 0.05, **p < 0.01.

For a schematic diagram of FC at no-go trials, see **Figure 3**. Compared to the HC group, the MDD group demonstrated higher connectivity between *l*M1 and preSMA (*t* = 3.214, *p* = 0.002, FDR corrected) (**Figure 4A**), and showed reduced connectivity between *l*M1 and *r*IFG in the beta band (*t* = −2.405, *p* = 0.02, FDR corrected) (**Figure 4B**). In additional, we obtained the predictor “the FC between *l*M1 and preSMA in the beta band” (*p* = 0.027, the overall correctly specified group percentage was 76.1%) using binary logistic regression. The ROC analysis indicated the FC was further taken out to examine the correlation between the candidate predictors and cognitive function deficits (**Supplemental Figure 1**).

#### Differences of FC in the Low Gamma Band at No-Go Trials

Compared to the HC group, the MDD group exhibited lower connectivity between preSMA and *r*IFG in the low gamma band (*t* = −2.612, *p* = 0.012, FDR corrected) (**Figure 4C**).

### Correlations Between FC and Clinical Information

Since scholars recently reported strong covariation of symptoms, age and sex on neuroimaging phenotypes (39), the relationships between FC in the beta, gamma bands and clinical information (including demographic information [age, gender, illness duration], psychopathology [total HRSD scores, factor scores], and neurocognitive [behavior scores] variables) were also systematically explored.

**Figure 5:**
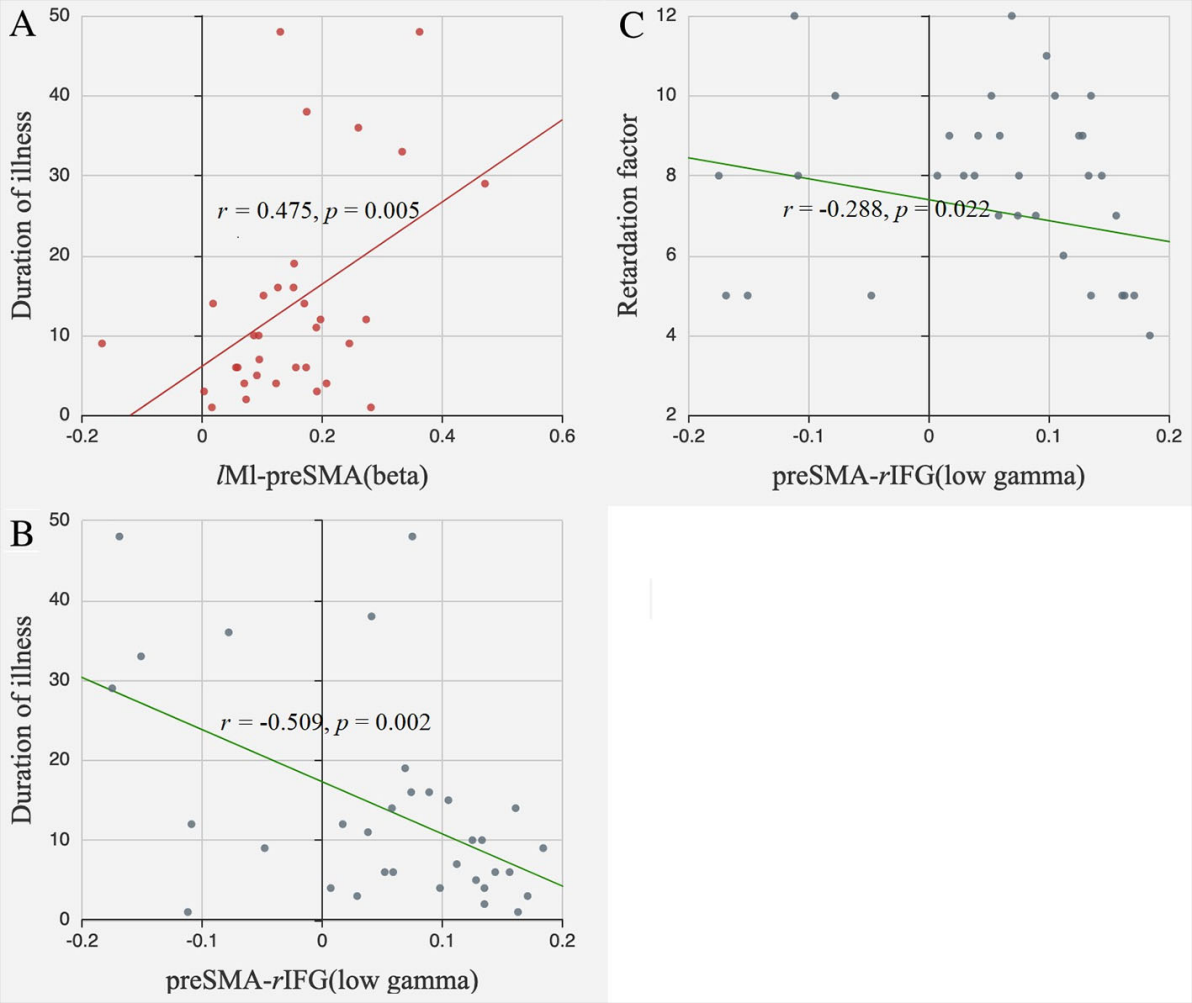
Correlations between FC and clinical information. **(A)** The FC between *l*M1 and preSMA in the beta band positively correlated with illness duration; **(B)** The FC between preSMA and *r*IFG in the low gamma band negatively correlated with illness duration; **(C)** The FC between preSMA and *r*IFG in the low gamma band negatively correlated with retardation factors.

The FC between *l*M1 and preSMA in the beta band was positively correlated with illness duration (*r* = 0.475*, p* = 0.005, FDR corrected). The FC between preSMA and *r*IFG in the low gamma band tended to be negatively correlated with illness duration (*r* = −0.509*, p* = 0.002, FDR corrected) and retardation factor scores (*r* = −0.288*, p* = 0.022, uncorrected) (**Figure 5**).

## Discussion

In this study, MDD patients exhibited alterations in frontal functional connectivity during response inhibition. The PS in *l*M1 and preSMA tended to be attenuated in the beta and gamma bands. Additionally, for the MDD group, there was an increased FC between *l*M1 and preSMA and a reduced FC between *l*M1 and *r*IFG in the beta band as well as a reduced FC between preSMA and *r*IFG in the low gamma band. In contrast, opposite patterns were observed in the HC group. Additionally, the FC between *l*M1 and preSMA in the beta band was associated with longer illness duration. Inversely, the FC between preSMA and *r*IFG in the low gamma band was related with shorter illness duration and was negatively correlated with retardation symptoms. This suggests that the activities of different frequency bands are related to cognitive deficits in depression.

Response inhibition refers to the important innate ability to cancel a planned movement when it is no longer required or potentially harmful. Response inhibition is commonly reported when a “stop” signal is used to cancel a planned movement (40). MDD profoundly alters perceptions and interactions with the proximate environment as well as impacting social environment, information and intellectual processing. Authors of previous studies have shown that this revocation may involve a right-lateralized cortices subcortical network (14, 41). However, when stopping is forewarned, more proactive inhibitory processes may be engaged (42). The importance of dissociating mood and cognitive symptoms has been highlighted in this study. It is believed that cognitive processes could underlie and enhance the negative thoughts that characterize depressive disorders. We suggest that changes in the frontal connectivity response inhibition could provide a clinically relevant potential pathological target for early phase experimental treatment studies with MDD patients.

### Altered PS Pattern in the MDD Group

The altered PS pattern in the *l*M1 and preSMA regions in MDD participants is related to inhibition deficits.

Scholars have previously demonstrated that beta-band activity has an anti-kinetic effect on motor performance whereas gamma-band activity is pro-kinetic in nature (43, 44). It was established that at 20 Hz (beta band), the human motor cortex tends to resonant activity and could entrain neuronal stimulations. Furthermore, there was a decline in beta-band activity before and during voluntary movement in the frontal motor cortex. This abnormality in beta oscillations in the fronto-parietal network could indicate a crucial piece of information about impaired ability in executive processing. However, a surge in gamma band activity has been detected before and during motor performance (43, 45) which also affected motor response time (46). It has been previously reported that gamma oscillations relied on GABAergic neuronal inhibition circuits (47, 48). The role of GABA may also be assessed in the human motor cortex during executive tasks (49). GABAB (49, 50) receptor-mediated pathways played a role in setting an inhibitory tone according to task context, while GABAA (51) receptor-mediated pathways could be modulated proactively with response certainty to optimize task performance.

There is converging evidence to suggest that the primary motor cortex (M1) is modulated during response inhibition given its role in shaping descending motor output (52). It has been found that the intercortical inhibitory networks within M1 had regulatory effects on descending commands that fine-tune movements in healthy people (53, 54). PreSMA and *r*IFG are crucial nodes for inhibitory control during stop-signal and go/no-go task. It is noteworthy that preSMA may result from a delay in the onset of inhibition on go-activation (42, 55).

In contrast with existing findings, we found that the PS in the beta and gamma bands decrease in M1, suggesting that an abnormally low activation of M1 may be closely related to pathological changes of GABA in MDD patients. It was reported that MDD patients had reduced GABA levels in their brains, decreased expression of GABAergic interneuron markers, as well as alterations in GABAA and GABAB receptor levels (56). Overall, M1 and preSMA may be affected by deteriorative changes in GABA receptor-mediated suppression of GABA release. Such an alteration may cause an imbalance of long-interval intracortical inhibition/short-interval intracortical inhibition (LICI/SICI) and aggravate changes to inhibition (44). In the present study, potential mechanisms within M1 and preSMA were identified that may support both proactive and reactive processes.

### Altered FC Pattern in the MDD Group

Analysis of the FC alterations in the MDD group suggests that the synergistic pattern of neural activity in the *l*M1, preSMA, and *r*IFG brain regions may be traits of MDD. In the HC group, response inhibition activated a distinct inhibitory process in the motor cortex. The interactions of both *r*IFG and preSMA with *l*M1 revealed similar temporal profiles during the no-go trials. This is in accordance with the increasing body of evidence which indicates that several areas are crucial for response inhibition, thus indicating that these regions work together to exert a causal control in the early phases of movement inhibition (53, 57).

#### FC in the Beta Band

It is possible that the no-go cues directly induce beta oscillation in these prefrontal areas, and that periodicity in the connected motor cortex is a natural consequence of this prefrontal oscillation. In past no-go trials studies (17, 18, 58), endogenous (top-down) inhibitory motor signals were transmitted in beta bursts in large-scale cortical networks. Beta oscillations might predominantly reflect endogenously driven processes and serve the maintenance of the status quo of a current sensory-motor or cognitive state (59). In previous report, impaired memory and attention efficiency abnormally enhanced beta activity containing more short-range frontal connections as well as inter-hemispheric temporo-parietal connections in people with depression. This adaptive compensatory mechanism is also reflected in the deterioration of flexibility in cognitive control (30).

Interestingly, we obtained similar results to previous authors who have suggested that the FC between preSMA and *l*M1 in the beta band in the MDD group was abnormally enhanced compared to the control group (6, 17, 59). In contrast, the FC between *r*IFG and *l*M1 was weakened in the MDD group. This reflects the attenuation of cortical circuits in an attempt to repair executive functions *via* (top-down) regulation from IFG.

#### FC in the Low Gamma Band

The outcome of a movement task which requires motor control may be improved by modulating the activity of both M1 and the sub-cortex region rather than M1 activity alone (43, 60). Authors of existing studies about the inhibitory control network in the human brain have repeatedly found that the activation of preSMA and *r*IFG is crucial for inhibitory control during stop-signal and go/no-go task. Analysis of converging evidence also suggests that both the preSMA and the *r*IFG are activated when preparing to stop but only the *r*IFG is activated when stopping (6, 17, 42, 53, 55). Consequently, scholars have identified the anterior SMA along with the *r*IFG as “negative motor regions” (61, 62).

Given the widely accepted view that brain oscillations are fundamental for communication between neuronal network elements, it could be predicted that the transmission of these inhibitory signals may be realized in rapid, periodic bursts of oscillatory brain activities within the prefrontal-central networks (that is, *r*IFG/M1 and/or preSMA/M1) at a distinct frequency. Proponents of a proposed “binding theory” suggest that neural populations in different cortical regions become synchronized with gamma-band oscillation, thereby strengthening the inter-cortical neural network (63). There is increased gamma activity in both regions, with preSMA preceding *r*IFG in healthy controls when preparing to stop (no-go stimuli) during the task. Early preSMA engagement may reflect the “setting up of inhibitory control” as well as *r*IFG monitors for the stop signal (gamma response). This information is then conveyed to the preSMA (coherent beta activity), which implements inhibitory control (beta response) (63–65). The response at the preSMA preceded that of the *r*IFG when preparing to stop.

In this study, we speculated that deceased FC between preSMA and *r*IFG in the low gamma band represented “the different activity pattern” in MDD. This could involve coherent beta activity in primary motor cortex and influence “negative motor regions” activity when preparing to stop. This is consistent with the theory that the preSMA plays a task-configuration function (that is, to prepare the brain's network to stop) while the *r*IFG is important for monitoring the need to stop and/or implement inhibitory control (53, 54). These findings could enable clinicians to discover cognitive deficits in people living with MDD earlier. Given that different patterns of FC provided objective imaging evidence, our findings could characterize potential damage to the brain areas (preSMA, *r*IFG) in depressed patients.

### Correlation Between FC and Depression Severity

We also found a positive correlation between the FC between *l*M1and preSMA in the beta band and illness duration. Meanwhile, in the low gamma band, the FC between preSMA and *r*IFG was negatively correlated with illness duration and retardation symptoms.

#### Duration of Illness

Duration of illness (DI) appears to be a negative factor for mood disorders. Long duration of mood disorders has been associated with lower treatment responses, increased suicidal risk, and cognitive deficits (66). Multivariate analyses revealed that a family history of psychiatric conditions positively correlated to DI (67). Authors of previous studies have found that dependency of brain wave connectivity patterns on psychiatric disease duration may be partially explained by differences in inhibitory behavior (68–71).

As detailed above, the FC between *l*M1 and preSMA in the beta band is a robust result of our study, independent of disease course duration. This may represent a valid trait-marker for depression. We suggest that there is a relationship between DI and the FC betweeen *l*M1 and preSMA in the beta band in the occurrence and development of cognitive deficits in people with MDD. However, it remains unclear why functional abnormalities of the ROIs are associated with illness duration but not MDD symptom severity.

Additionally, we found the FC between preSMA and *r*IFG in the low gamma band was negatively correlated with DI, suggesting that the severity of abnormal FC may be aggravated with disease progression. While correlations between the alterations of FC and DI were found, we could not tell whether functional abnormality is a result of illness development or a factor which contributes to the occurrence and development of depressive symptoms.

#### Retardation Symptoms

MDD patients tend to exhibit significantly low mood and poor motivation in addition to severe retardation symptoms. It is hard for these people to concentrate, make decisions, or quickly refuse. In the current study, we found that the FC in “negative motor regions” in the low gamma band was negatively correlated with illness duration. Meanwhile, the FC was also negatively correlated with retardation factor scores. The previous study concluded that the gamma wave is mainly involved in the evaluation of subjective uncertainty, or conflict about the current information (31). This can explain why patients with depression are more likely to be trapped in inner self-directed top-down conflicting thinking, resulting in a reduction in behavior and cognitive flexibility. Therefore, these findings could provide a neurophysiological explanation for ruminative processes and retardation symptoms.

### Future Insight—the Predictive Value of FC

We found that specific abnormal patterns of PS and FC could serve as a neuroelectrophysiology target for future treatment for people living with MDD. Many scholars have described that pre-treatment aberrant connectivity pattern is altered following treatment in MDD patients (72). Additionally, a reduced FC between preSMA and *r*IFG in the low gamma band has been reported following antidepressant treatment, indicating the valuable role of altered FC as a biomarker of efficient treatment (54). Authors of neuroimaging studies have demonstrated that non-invasive brain stimulation techniques such as TMS (73–75), tDCS (76, 77), and cTBS (78, 79) altered neural activation within the *r*IFG or preSMA and could affect inhibitory control in a positively or negatively.

Artificial modulation of the oscillatory activity of beta bands in the motor-related area of the brain has recently been investigated to improve motor performance (43). When transcranial alternating current stimulation (tACS) was applied to M1, there was an attenuation of finger movement velocity (58) and force (18) at beta-band frequencies (“beta tACS”) while at gamma-band frequencies (“gamma tACS”), tACS increased finger movement velocity and force (18, 80, 81). Moreover, participants performed better on a visuomotor tracking task when tACS was directed to M1 and Cz areas at 80 Hz (82).

A response may be evoked by a decrease in the activation threshold prompted by a reduction in LICI. Based on our findings, we suggest that LICI set a general inhibitory tone relative to response expectations, whereas SICI is modulated until a response decision is taken. Potential mechanisms have been previously identified within M1 which may support both proactive and reactive processes (44). In the future, rTMS can be employed to adjust alterations in LICI (GABAb-R) and SICI (GABAa-R) and thus normalize M1 for inhibition. Therefore, our researcher team is now focusing on artificially modulating oscillatory activity in the beta and gamma bands of the motor-related area of the brain to improve performance.

## Limitations

The limitations of the current work are as follows. First, our study was conducted with a small cohort of participants living with MDD. Second, the relationship between the direction of regulation among *l*M1, preSMA, and *r*IFG regions were not considered. Third, a multi-dimensional combination of indicators, for example electrophysiological, neurocognitive, and neuroimaging measures, might be more sensitive and robust for recognizing cognitive deficits and predicting treatment efficacy.

## Conclusion

In conclusion, aberrant frontal functional connectivity was found during response inhibition in MDD patients. Lower beta and gamma bands activities were found in *l*M1 and preSMA. A longer duration of illness was linked with the FC between *l*M1 and preSMA in the beta band while a shorter DI and retardation symptoms were associated with the FC between preSMA and *r*IFG in the low gamma band. This demonstrated changes in frontal connectivity response inhibition which could lead to cognitive deficits in depression. DI is central to the functional deficits pertaining to response inhibition in MDD. These results could serve as a potential pathological target for those conducting clinical trials in the future.

## Data Availability Statement

The raw data supporting the conclusions of this article will be made available by the authors, without undue reservation.

## Ethics Statement

The studies involving human participants were reviewed and approved by the ethics committee of Nanjing Brain Hospital Affiliated to Nanjing Medical University. The patients/participants provided their written informed consent to participate in this study.

## Author Contributions

YLH, QL, and ZJY designed experiments; YLH, MR, PHL, HLZ, and HFW carried out experiments; YLH, PHL, and HLZ analyzed experimental results. ZPD analyzed sequencing data and developed analysis tools. ZPD and YLH wrote the manuscript.

## Funding

This work was supported by the National Natural Science Foundation of China (81571639, 81871066); the Jiangsu Provincial Key Research and Development Program (BE2018609); a Jiangsu Provincial Medical Innovation Team grant for the Project—Invigorating Healthcare through Science, Technology and Education (CXTDC2016004); the Nanjing Science and Technology Development Plan (YKK15106, YKK15110, YKK16146); a Jiangsu Provincial Medical Youth Talent grant for the Project—Invigorating Healthcare through Science, Technology and Education (QNRC2016049, QNRC2016050); the Science and Technology Program of Nanjing, China (201,605,039), and the Nanjing Medical Science and Technique Development Foundation (QRX17178).

## Conflict of Interest

The authors declare that the research was conducted in the absence of any commercial or financial relationships that could be construed as a potential conflict of interest.
