# Supplementary material for: Connectivity of the Frontal Cortical Oscillatory Dynamics Underlying Inhibitory Control During a Go/No-Go Task as a Predictive Biomarker in Major Depression
Source: Front Psychiatry. 2020 Aug 3;11:707. doi: 10.3389/fpsyt.2020.00707 (PMC7416643; doi:10.3389/fpsyt.2020.00707)
Supplement: Supplementary file 1 [file Image_1.pdf]

we got the predictor "the FC between *l*M1 and preSMA in the beta band" (p = 0.027, the overall correctly specified group percentage was 76.1%) by binary logistic regression analysis. The Receiver Operator Curve (ROC) analysis indicated that the FC between *l*M1 and preSMA in the beta band (AUC = 0.699, p <0.05, specificity 57.1% and sensitivity 90.6%) may supply a clue to help differentiate disinhibition between depressed patients and healthy controls.

In previous report, abnormally enhanced beta activity about impaired memory and attention containing more short-range frontal connections as well as inter-hemispheric temporoparietal connections in depressed patients. This mechanism is also reflected in the deterioration of flexibility in cognitive control [30]. So the ROC is constructed for investigating the discriminating FC to potentially distinguish cognitive deficits in MDD from HC (Supplementary Fig.1).

Supplementary Fig. 1: Receiver Operator Curve (ROC) for results of a potential discriminant analysis on disinhibition

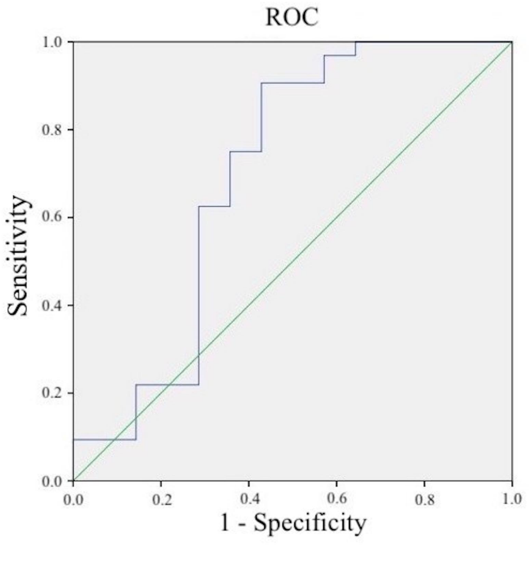

The ROC for results of a discriminant analysis on disinhibition

The ROC shows the specificity (57.1%) and sensitivity (90.6%) for disinhibition in MDD, with an area under the curve of 0.699.
